# A multicentre study of the evidence for customized margins in photon breast boost radiotherapy

**DOI:** 10.1259/bjr.20150603

**Published:** 2015-12-28

**Authors:** Emma J Harris, Mukesh B Mukesh, Ellen M Donovan, Anna M Kirby, Joanne S Haviland, Raj Jena, John Yarnold, Angela Baker, June Dean, Sally Eagle, Helen Mayles, Claire Griffin, Rosalind Perry, Andrew Poynter, Charlotte E Coles, Philip M Evans, On behalf of the IMPORT high trialists

**Affiliations:** ^1^ Radiotherapy and Imaging, Institute of Cancer Research Sutton, UK; ^2^ Oncology Centre, Cambridge University Hospital NHS Foundation Trust, Cambridge, UK; ^3^ Oncology Centre, Colchester Hospital University NHS Trust, Colchester, UK; ^4^ The Breast Unit, Royal Marsden Hospital NHS Foundation Trust, Sutton, UK; ^5^ ICR-CTSU, Institute of Cancer Research, Sutton, UK; ^6^ Macmillan Survivorship Research Group, Faculty of Health Sciences, University of Southampton, UK; ^7^ Department of Physics, The Clatterbridge Cancer Centre NHS Foundation Trust, Wirral, UK; ^8^ Department of Radiotherapy, Ipswich Hospital NHS Trust, Ipswich, UK; ^9^ Radiotherapy Department, Peterborough City Hospital, UK; ^10^ CVSSP, University of Surrey, Guildford, UK

## Abstract

**Objective:**

To determine if subsets of patients may benefit from smaller or larger margins when using laser setup and bony anatomy verification of breast tumour bed (TB) boost radiotherapy (RT).

**Methods:**

Verification imaging data acquired using cone-beam CT, megavoltage CT or two-dimensional kilovoltage imaging on 218 patients were used (1574 images). TB setup errors for laser-only setup (*d*_laser_) and for bony anatomy verification (*d*_bone_) were determined using clips implanted into the TB as a gold standard for the TB position. Cases were grouped by centre-, patient- and treatment-related factors, including breast volume, TB position, seroma visibility and surgical technique. Systematic (Σ) and random (σ) TB setup errors were compared between groups, and TB planning target volume margins (*M*_TB_) were calculated.

**Results:**

For the study population, *Σ*_laser_ was between 2.8 and 3.4 mm, and *Σ*_bone_ was between 2.2 and 2.6 mm, respectively. Females with larger breasts (*p* = 0.03), easily visible seroma (*p* ≤ 0.02) and open surgical technique (*p* ≤ 0.04) had larger *Σ*_laser_. *Σ*_bone_ was larger for females with larger breasts (*p* = 0.02) and lateral tumours (*p* = 0.04). Females with medial tumours (*p* < 0.01) had smaller Σ_bone_.

**Conclusion:**

If clips are not used, margins should be 8 and 10 mm for bony anatomy verification and laser setup, respectively. Individualization of TB margins may be considered based on breast volume, TB and seroma visibility.

**Advances in knowledge:**

Setup accuracy using lasers and bony anatomy is influenced by patient and treatment factors. Some patients may benefit from clip-based image guidance more than others.

## INTRODUCTION

Cancer recurrence within the breast is most likely to occur in the region of the tumour bed (TB). A radiotherapy (RT) boost to the TB reduces the risk of local relapse and is recommended for patients at higher risk of recurrence.^1^ It has also been shown that an RT boost to the TB can increase the risk of normal tissue toxicity such as fibrosis.^2^ The risk of fibrosis may increase as the volume of the TB planning target volume (PTV) increases.^3^ A larger PTV may also affect the dose delivered to other normal tissues. For example, recent work by Darby et al^4^ suggests there is no safe dose threshold for cardiac tissues. A suitable boost PTV margin will encompass the TB throughout the course of RT and treat minimal non-target tissue to reduce the risk of both local relapse and normal tissue toxicity.

Titanium surgical clips and gold fiducial markers have been shown to be effective imaging surrogates for the TB.^5,6^ Here, we refer to both surgical clips and gold markers as clips. TB clips can influence placement of fields^7^ and assist in the planning of partial breast and boost RT.^8^ Increasingly, photon boosts are used as it is easier to visualize and optimize planned dose distribution compared with electron boosts. Combining photon boost and TB clips enables the use of image-guided RT to verify the position of the TB. It has been shown that using clips, PTV margins of 5 mm can be used safely to deliver both sequential and synchronous photon boost RT with steep dose gradients.^9^ Clip-based image-guided RT and 5-mm PTV margins are strongly recommended by the Intensity Modulated Partial Organ Radiotherapy (IMPORT) trials group.^7–9^ However, this is not routine practice worldwide. A common alternative imaging verification method is X-ray (megavoltage or kilovoltage) imaging of bony anatomy, and if imaging is not available, a laser-based setup using skin marks is used. Neither X-ray imaging using bony anatomy nor laser setup can directly verify the position of the TB in the absence of implanted markers. This is because the breast can move independently from the chest wall and the TB may change in shape and size within the breast, *e.g.* reabsorption of the TB seroma fluid.

This study aimed to investigate the consequences of using laser-only verification or bony anatomy verification on setup accuracy in TB boost RT. The study used imaging data from five UK IMPORT High trial centres.^10^ These data were from kilovoltage cone-beam CT (kVCBCT), megavoltage CT (MVCT) and two-dimensional kilovoltage (2DkV) planar imaging. Analysis involved matching of clips and bony anatomy to reference images. The study investigated:

1. TB setup errors for (i) bony anatomy verification and (ii) laser-based setup, using TB clip position as the gold standard TB position.
2. Influence of patient-, surgery- and RT-related factors on TB setup errors, including breast volume, position of the TB, the presence of seroma, surgical technique, the presence of posterior fascia clip(s), number of clips, time from surgery to CT, time from CT to RT and trial arm.
3. Time required to match verification images with reference images to bony anatomy and clips.

## METHODS AND MATERIALS

National Health Service Research Ethics Committee (REC) approval for this study was granted as a substantial amendment to IMPORT High National Health Service REC approval (Cambridgeshire 4 REC on 22/10/2010 (REF: 08/H0305/13)). All IMPORT High patients consented for their imaging and planning data to be used for research.

### Patients

218 patients, from 5 cancer centres were included (Centres A–E). All patients received whole breast RT and TB boost as part of the UK IMPORT High trial (testing sequential *vs* synchronous integrated boost).^10,11^ Patients consented for their data to be used for research purposes. All patients had surgical clips implanted into the TB and were treated using clip-based verification (using online or offline verification protocols) for their TB boost.^5^ This was a retrospective study, which had no impact on the patients' treatment. Patients were selected sequentially, by the date of their treatment.

### Patient setup and imaging

All patients were positioned using laser alignment of tattoos. Two or three tattoos were marked: one anterior, medial at the midline and one or two lateral. All centres used an immobilization wedge beneath the knees, centre B used ankle immobilization also, and all patients were treated in supine position using a breast board with either one or two arms abducted.

All patients had CT imaging for treatment planning. At treatment, patients were initially positioned using lasers (laser setup) and then imaged using either kVCBCT (Synergy, Elekta Ltd, UK) (Centre A, *n* = 79), MVCT (TomoTherapy, Accuray Inc., Sunnyvale, CA) (Centre B, *n* = 39) or orthogonal (0° and 90°) 2DkV fields (OBI Varian Oncology Systems Inc., Paolo Alto, CA) (Centres C, D and E, *n* = 40, 30 and 30, respectively). For Centre A, using an offline protocol, the mean number of images acquired was 5.2 for control arm patients (sequential boost) and 7 for test arm patients (synchronous boost). For centres an online protocol (B–E), the number of images acquired was 8 and 15 for control and test arm patients, respectively.

### Imaging data analysis

**Figure 1.**
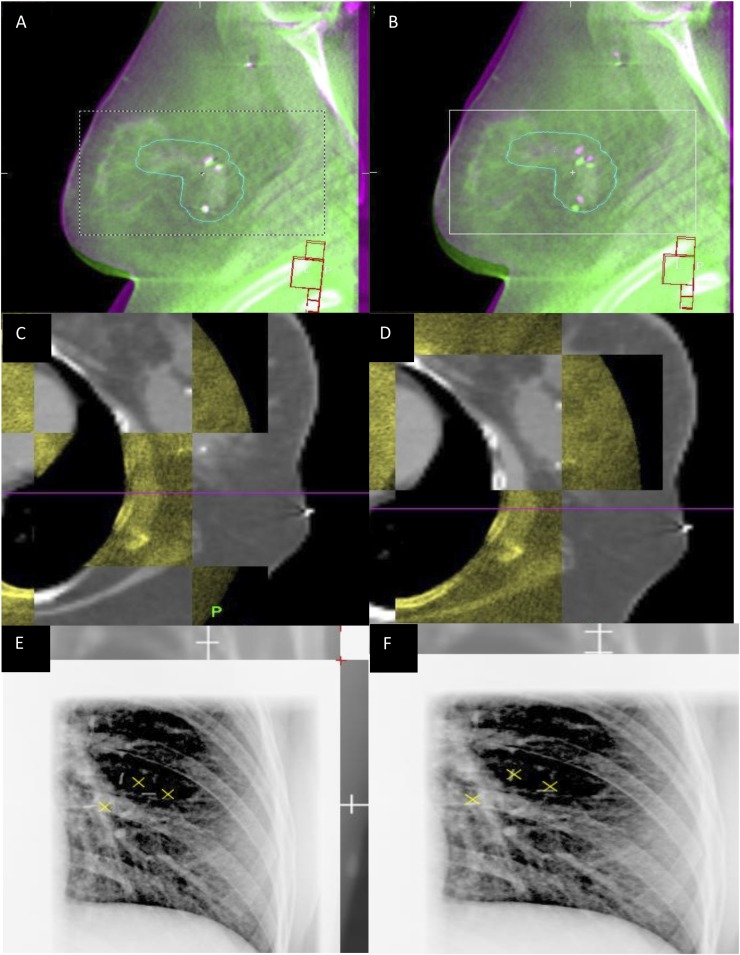
Example images used for setup error analysis. (a) and (b) show sagittal planning CT images (pink) overlaid with sagittal cone-beam CT images (green). Figures (c) and (d) show planning axial CT images in grey and axial megavoltage CT in yellow. Figures (e) and (f) show two-dimensional kilovoltage projection images overlaid on digitally reconstructed images from the planning CT; planning CT clips positions are marked with yellow crosses. Images in the left-hand column (a, c and e) show bone-matched images and images in the right hand column (b, d and f) show clip-matched images. For colour image see online.

All image data analysis for this study was performed offline. For each image, matching of the reference and verification images was performed using^1^ clip match and^2^ bony anatomy match (Figure 1). Clip match gave the translational shift between clip position after laser set-up and the reference clip position (on planning CT). Bony anatomy match gave the translational shift between bony anatomy position after laser set-up and the reference bony anatomy position (on planning CT). Shifts in the left–right (LR), superior–inferior (SI) and anteroposterior (AP) directions were recorded. The time to perform the clip and bone matches was recorded. One or two observers performed the matching of all images at each centre (Centre A, EH; Centre B, MM; Centre C, AB; Centre D,  EH; and Centre E, EH and RP) and were blinded to image matches recorded during treatment.

Interobserver error analysis was carried out by three observers who matched three images from three patients selected at random, at Centres A (CBCT), B (MVCT) and C (2DkV). Mean setup error across observers was calculated per image, and the difference between each observer's measurement and mean was determined. Interobserver error was the standard deviation in differences, calculated for each imaging technique. For intraobserver analysis, three observers, EH (CBCT and 2DkV), MM (MVCT) and AB (2DkV), were asked to match three images on three different days. Mean setup errors across repeat measurements were calculated per image, and the difference between each observer's measurement and mean was determined. The intraobserver error was the standard deviation in differences calculated for each observer.

### Tumour bed setup errors and margins

TB setup error after laser-based setup was the distance between the position of the TB clips after laser setup and the reference TB clip position, *i.e.* TB clip position was used as the gold standard for TB position. This was referred to as *d*_laser_ and was the TB setup error if no imaging verification was used. TB setup errors after bony anatomy verification were the distance between TB position after bony anatomy match and the reference TB position. This was referred to as *d*_bone_ and was the TB setup error if imaging verification of bony anatomy was performed and the patient was shifted to ensure bony anatomy position was correct. An individual patient's systematic and random setup errors, for laser and bony anatomy verification, were calculated using the mean and root mean square of *d*_laser_ and *d*_bone_ using all images available for the patient. The group systematic TB setup error for laser setup (*Σ*_laser_) and bony anatomy verification (*Σ*_bone_) and the group random TB setup error for laser setup (*σ*_laser_) and bony anatomy verification (*σ*_bone_) were calculated following refs.^9^ and.^12^ For bony anatomy verification, TB setup errors are for an online imaging protocol with no action level. A TB PTV margin (M_TB_) formulation for breast boost was used to estimate the tumour bed margin required for laser setup and bony anatomy verification:^13^

(1) $$M_{TB}=2.5\sum +0.3\sigma ,$$

To estimate M_TB_, setup errors were added in quadrature with the errors associated with using clips as a surrogate for the TB. TB surrogate systematic and random errors were 1.2 and 0.9 mm, respectively, based on the findings of.^14^

### Patient- and treatment-related factors

**Figure 2.**
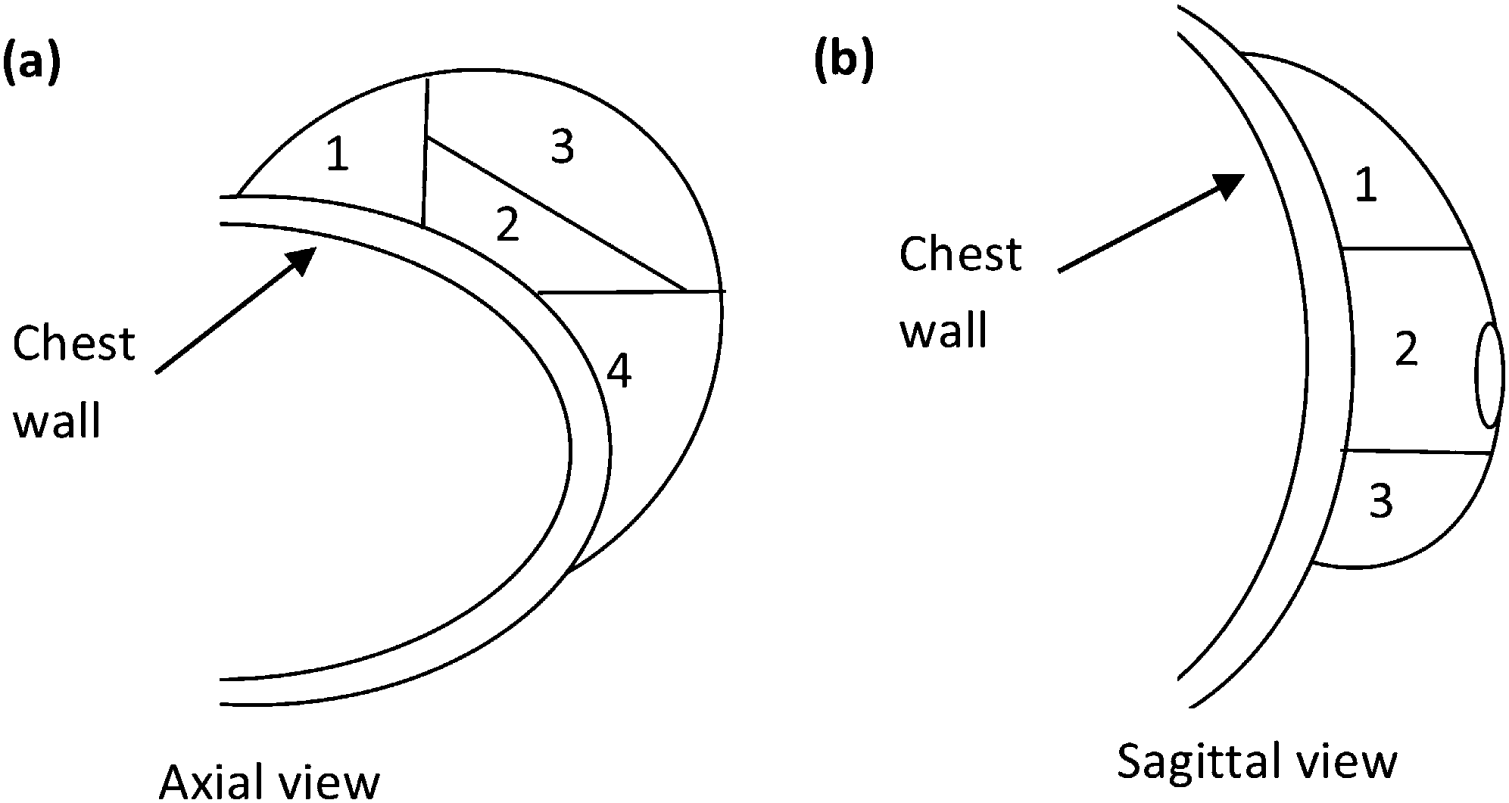
Schematic diagram showing (a) tumour bed (TB) position viewed on axial CT slice (1 =medial, 2 =chest wall, 3 =anterior and 4 =lateral) and (b) TB superior–inferior position viewed on sagittal CT slice (1 = superior, 2 = middle and 3 = inferior).

Patient and treatment factors were collected (Table 1). Patient-related factors included breast volume (whole-breast PTV constrained by skin surface and chest wall) and TB position (Figure 2). Factors relating to patients' surgery included apposed (closed) or unapposed (open) cavity, the latter allowing seroma fluid to accumulate. Seroma visibility was scored by a single radiation oncologist (MM), who rated seroma as not visible/subtle or easily visible^15^ and determined the number of clips placed at the posterior fascia and in the excision cavity. RT-related factors were days between CT and RT (*t*_CT–RT_), days between surgery and RT (*t*_Surgery–RT_) and trial arm.

**Table 1.** Patient and treatment factors

Variables	Number of patients with data in each group	Total number of patients with data	Median value (range)
**Patient related:**			
TB axial position (1/2/3/4) (Figure 1.)	30/96/33/59	218	
TB SI position (1/2/3) (Figure 1.)	107/90/21	218	
Breast volume (above median/below median) (cm^3^)	109/109	218	855 (118–2847)
**Surgery related:**			
Seroma visibility (not visible/easily visible)	158/60	218	
Surgical closing technique (closed/open)	113/88	201	
Number of clips (above median/below median)	109/109	218	6 (4–14)
Clip in posterior fascia (no/yes)	40/178	218	
**Radiotherapy related:**			
Time from surgery to CT (days)	101/102	203	133 (32–481)
Time from CT to RT (days)	102/102	204	20 (3–112)
Trial arm [synchronous (test) or sequential (control)]	72/146	218	

RT, radiotherapy; SI, superior–inferior; TB, tumour bed.

Factors have been categorized according to the information they provide.

Median values and ranges are given for continuous variables.

### Statistical methods

Cases were grouped according to patient- and treatment-related factors. Cases were dichotomized above and below the median value for breast volume, number of clips, time from surgery to planning CT (*t*_S–CT_) and time from planning CT to RT (*t*_CT–RT_). Additionally, cases were grouped according to TB position, seroma visibility, surgical closing technique, the presence of clip in the posterior fascia and trial arm (synchronous or sequential boost).

All data were tested for normality using Shapiro–Wilks test, and results indicated that the majority of the data (90%) were non-normal. Differences between median *d*_laser_ and *d*_bone_ and differences between centres were tested using Wilcoxon and Kruskal–Wallis tests.

Differences in systematic and random TB setup errors between (i) techniques (laser setup and bony anatomy verification), (ii) centres and (iii) between groups by patient- or treatment-related factors were tested. Non-parametric Levene's test was used to test for differences in the variance of patient systematic *d*_laser_ and *d*_bone_. Kruskal–Wallis test was used to test for differences in the patients' random *d*_laser_ and *d*_bone_. Relationships between variables shown to give significantly different systematic errors were investigated using Kruskal–Wallis tests. For factors with two or more groups, sensitivity analysis was performed by removing one group at a time and repeating tests using Holms–Bonferroni correction.

## RESULTS

### Tumour bed setup errors and margins

Unless otherwise stated, all differences were statistically significant, and *p*-values were <0.001. The number of patients and images (fractions) analysed for each centre is given in Table 2. At Centres A and C, all available images were analysed. At Centres B, D and E, five, six and six images per patient were analysed, respectively. Using only five images was validated by a comparison of setup data calculated using 15 images *vs* 5 images for 28 cases. The mean differences in patients' mean and standard deviation of setup errors were 0.006 and 0.013 cm, respectively.

**Table 2.** Mean (and 95th percentile) tumour bed (TB) setup errors for laser-based setup (d_laser_) and bony anatomy imaging verification (*d*_bone_), in the left–right (LR), superior–inferior (SI) and anteroposterior (AP) directions, and three-dimensional (3D) vector magnitude and image-matching times

Centre	Number of patients	Total number of fractions analysed	Absolute TB setup errors for laser setup (*d*_laser_) Mean (95th percentile) (mm)				Absolute TB setup errors for bony anatomy (*d*_bone_) Mean (95th percentile) (mm)				Mean time (STD) (s)	
			LR	SI	AP	3D	LR	SI	AP	3D	*t*_bone_	*t*_clip_
ALL	218	1574	3.1 (8.0)	2.9 (7.8)	3.9 (11.1)	5.7 (13.1)	2.0 (6.0)	2.6 (8.0)	2.1 (6.3)	4.1 (9.8)	73 (49)	66 (45)
A (kVCBCT)	79	504	2.9 (6.8)	2.8 (7.2)	3.4 (8.4)	5.1 (10.2)	1.9 (5.5)	2.4 (5.9)	2.2 (6.6)	3.9 (8.5)	**29 (10)**	**96 (30)**
B (MVCT)	40	200	**3.9 (9.7)**	**3.6 (8.9)**	**8.5 (18.1)**	**9.9 (19.4)**	**1.4 (4.5)**	**1.3 (6.0)**	**1.8 (5.9)**	**3.0 (7.3)**	**122 (37)**	**110 (35)**
C (2DkV)	39	510	3.0 (8.0)	2.9 (7.2)	**2.6 (7.0)**	4.6 (9.1)	2.3 (7.0)	2.9 (10.0)	2.0 (6.0)	4.3 (11.0)	**39 (18)**	**21 (12)**
D (2DkV)	30	180	2.6 (7.7)	2.9 (8.0)	3.2 (9.0)	5.1 (11.0)	2.1 (6.0)	3.3 (9.0)	2.2 (7.0)	4.6 (9.9)	**88 (25)**	**32 (10)**
E (2DkV)	30	180	**3.9 (10.0)**	3.0 (7.0)	**5.0 (12.3)**	6.6 (13.4)	2.0 (5.0)	3.1 (8.0)	2.3 (7.3)	4.5 (10.1)	**105 (50)**	**45 (27)**

kVCBCT, kilovoltage cone-beam CT; MVCT, megavoltage CT; STD, standard deviation; t_bone_, time to match images using bony anatomy; t_clip_, time to match images using clip; 2DkV, two-dimensional kilovoltage.

Values given in bold indicate significant differences (p ≤ 0.05) between centres.

Intraobserver and interobeserver errors were <1.4 mm for all imaging modalities. There were no significant differences in observer errors between centres (*p* = 0.34).

Mean (and 95th percentile) absolute values of *d*_laser_ and *d*_bone_ in the LR, SI and AP directions are given in Table 2. Over all data, the mean absolute TB setup error for laser-only setup (*d*_laser_) and for bony anatomy verification (*d*_bone_) was <4 and 3 mm in all directions, respectively. Compared with other centres, mean *d*_laser_ and *d*_bone_ was significantly greater and smaller in all directions for Centre B (MVCT), respectively. Variation between centres was greatest in the AP direction. *d*_laser_ was statistically significantly greater than *d*_bone_ in all directions across all centres.

Group systematic (*Σ*) and random (*σ*) errors for laser setup and bone verification are given in Table 3. Combining the data from all centres, *Σ*_laser_ was statistically significantly greater than *Σ*_bone_ in the LR and AP directions but not in the SI direction. Centre B had smaller *Σ*_bone_ compared with other centres in all directions and had larger *Σ*_laser_ compared with other centres (*p* = 0.002). TB margins for laser setup and bony anatomy verification are given in Table 4.

**Table 3.** Systematic and random tumour bed (TB) setup errors for laser-only setup and bone verification for each centre and all centres combined

Centre	Laser setup random error σ_laser_ (mm)			Laser setup systematic error (Σ_laser_) (mm)			Bone verification random error (σ_bone_) (mm)			Bone verification systematic error (Σ_bone_) (mm)		
	LR	SI	AP	LR	SI	AP	LR	SI	AP	LR	SI	AP
ALL	3.3	2.9	3.3	3.1	2.8	3.4	1.9	2.8	2.3	2.2	2.6	2.2
A (kVCBCT)	2.7	3.0	2.7	2.8	2.4	2.9	**1.6**	2.9	2.4	2.3	2.2	2.2
B (MVCT)	**4.4**	3.2	**4.7**	3.3	2.7	**4.4**	**1.7**	**2.2**	2.5	**1.1**	**1.6**	**1.4**
C (2DkV)	3.2	2.5	2.5	3.0	2.8	2.7	2.2	3.4	2.3	2.5	2.6	2.4
D (2DkV)	2.6	3.0	3.1	2.5	2.8	2.7	2.0	2.6	2.1	2.1	2.9	2.4
E (2DkV)	4.1	2.6	3.5	3.7	2.7	**3.6**	2.1	2.7	2.1	2.2	2.9	2.1

AP, anteroposterior; ; kVCBCT, kilovoltage cone-beam CT; LR, left–right; MVCT, megavoltage CT; SI, superior–inferior; 2DkV, two-dimensional kilovoltage.

Values given in bold indicate significant differences (p ≤ 0.05) between centres.

**Table 4.** Tumour bed planning target volume margins (*M*_TB_) or all centres combined

Laser setup *M*_TB_ (mm)			Bone verification *M*_TB_ (mm)		
LR	SI	AP	LR	SI	AP
9.0	9.0	10.0	7.0	8.0	7.0

AP, anteroposterior; LR, left–right; SI, superior–inferior.

### Association of tumour bed setup errors with patient- and treatment-related factors

Breast volume, seroma visibility and surgical technique were found to influence *Σ*_laser_ (Table 5). Females with larger breasts (*p*=0.03), easily visible seroma (*p*-values ≤ 0.02) and who have received an open surgical closing technique (*p*-values ≤ 0.04) had larger *Σ*_laser_. Breast volume and TB axial position were found to influence *Σ*_bone_ (Table 5). *Σ*_bone_ was larger in one direction for females with larger breasts (*p* = 0.015) and lateral tumours (*p* = 0.04). Females with medial tumours (*p* = 0.002) had smaller *Σ*_bone_. No statistically significant associations between breast volume, TB position, seroma visibility and surgical closing technique were found.

**Table 5.** Systematic tumour bed (TB) setup errors for laser setup (Σ_laser_) and for bone verification (Σ_bone_) for groups determined using patient- and treatment-related factors

Laser	Factor	Group 1	**Σ**_laser_ (mm)	Group 2	**Σ**_laser_ (mm)	*p*-value	Direction
	Breast volume	<855 cm^3^	2.5	≥855 cm^3^	4.2	0.03	SI
	Seroma visibility	Not visible/subtle	2.8	Easily visible	3.5	0.02	LR
		Not visible/subtle	2.6	Easily visible	3.2	0.002	SI
		Not visible/subtle	3.1	Easily visible	4.1	0.005	AP
	Surgical closing technique	Closed	2.7	Open	3.3	0.02	LR
		Closed	2.5	Open	3.2	0.04	SI
**Bones**	**Factor**	**Group 1**	**Σ_bone_(mm)**	**Group 2**	**Σ_bone_(mm)**	***p*-value**	**Direction**
	TB axial position	1, 2 and 3	2.1	4	2.7	0.04	LR
	TB axial position	1	1.6	2, 3 and 4	2.3	0.002	AP
	Breast volume	<855 cm	1.9	≥855cm^3^	2.7	0.015	SI

AP, anteroposterior; LR, left–right; SI, superior–inferior; TB, tumour bed.

*P*-values for univariate non-parametric Levene's test are given. Data given only for factors that gave a significant difference in systematic bony anatomy verification error between patient groups (*p* < 0.05).

Random TB setup errors (Table 6) for laser setup (*σ*_laser_) were influenced by breast volume and seroma visibility. Random TB setup errors for bony anatomy verification (*σ*_bone_) were influenced by TB axial position, breast volume, surgical closing technique and trial arm (*p*-values < 0.05).

**Table 6.** Random tumour bed (TB) setup errors for laser setup (σ_laser_) and for bone verification (σ_bone_) for groups determined using patient- and treatment-related factors

Laser	Factor	Group 1	σ_laser_ (mm)	Group 2	σ_laser_ (mm)	*p*-value	Direction
	TB PTV volume	<39.5 cm^3^	2.6	≥39.5 cm^3^	2.8	0.041	LR
	TB PTV volume	<39.5 cm^3^	2.6	≥39.5 cm^3^	3.1	0.023	AP
	Breast volume	<855 cm	2.4	≥855 cm^3^	3.1	0.006	LR
	Breast volume	<855 cm	2.4	≥855 cm^3^	2.8	0.02	SI
	Seroma visibility	Not visible/subtle	2.6	Easily visible	3.1	0.034	AP
**Bones**	**Factor**	**Group 1**	**σ_bone_ (mm)**	**Group 2**	**σ_bone_ (mm)**	***p*-value**	**Direction**
	TB axial position	1	2.0	2, 3and 4	1.6	0.025	LR
	TB axial position	1	1.5	2, 3 and 4	2.1	0.004	AP
	TB SI position	1	1.8	3	1.1	0.006	LR
	Breast volume	<855 cm	2.2	≥855 cm^3^	2.6	0.007	LR
	Breast volume	<855 cm	1.7	≥855 cm^3^	2.3	0.002	SI
	Trial arm	Synchronous	2.1	Sequential	1.8	0.01	AP
	Surgical technique	Closed	1.4	Open	1.9	<0.001	LR
	Surgical technique	Closed	2.3	Open	2.6	0.009	SI

AP, anteroposterior; LR, left–right; PTV, planning target volume; SI, superior–inferior, TB, tumour bed.

*P*-values for univariate Kruskal–Wallis test are given.

Data given only for factors that gave a significant difference in systematic bony anatomy verification error between patient groups (*p* < 0.05).

The difference in combined timing data for matching using bony anatomy and clips was not statistically significant (*p* = 0.29). Within individual centres, the time to match images using bony anatomy (*t*_bone_) and clips (*t*_clip_) was different except for in Centre B. There was a statistically significant difference between matching times between all centres except between Centres D and E. The time required to analyse MVCT images was greatest.

## DISCUSSION

### Tumour bed setup errors and margins

TB setup errors using laser setup were slightly larger than those of bony anatomy verification. This study found the mean three-dimensional *d*_bone_ (magnitude of the 3D vector for *d*_bone_) to be 4.1 mm, smaller than that reported in previous studies on small cohorts (*n* < 12) with median of 5.4 mm^18^ and mean of 6 mm.^19^ Although our results differ from these smaller studies, they are in keeping with a larger study by Penninkhof et al (*n* = 80)^16^ who found *Σ*_laser_ to be 2.6 mm (LR), 2.5 mm (SI) and 3.4 mm (AP). Penninkhof et al also evaluated the systematic error after an offline 2D portal imaging protocol and found systematic error Σ_bone_ of 2.3 mm (LR), 2.4 mm (SI) and 2.8 mm (AP), which were similar to values of *Σ*_bone_ in the present study.

### Variation in tumour bed setup errors between centres

There were small but statistically significant differences in absolute TB setup errors between centres. These were greatest in the AP direction. At Centre E, the cause was unknown and was investigated. At Centre B, a non-zero mean systematic mean error was due to couch sag, discussed in a previous report,^20^ which introduced the large mean absolute errors (Table 2) and overall systematic error (Table 3). Both Centres B and E used an online imaging protocol, which will remove these errors. Best practice is to eliminate such errors.

Centre B had smaller *d*_bone_ in all directions. The poorer imaging resolution of MVCT and higher X-ray energy made MVCT matching less straightforward^20^ and is evident from longer matching times (Table 2). Poorer visibility of landmarks, making it harder to match images, may have accounted for the smaller difference between clips and bony anatomy at Centre B. Poorer image quality was proposed as a contributing factor to smaller estimated setup errors using megavoltage compared with kilovoltage imaging.^21^ Exclusion of centre B in the overall calculation of 3D TB setup error for bony anatomy verification gave 3D *d*_bone_ = 4.8 mm, which is closer to values reported in^18^ and.^19^

### Influence of patient- and treatment-related factors on setup errors

Breast volume, seroma visibility and surgical closing technique affected TB systematic errors for laser setup. Changes in clip positions (relative to each other) over a course of RT may affect the accuracy of laser setup to skin marks. Penninkhof^16^ found patients with open surgical technique had greater clip motion compared with those with closed surgical technique, although the difference in motion was not significant (*p* = 0.22). Previously, we observed greater changes in clip positions in patients with large seroma.^22^

Axial TB position and breast volume affected TB systematic errors for bone verification. These factors and trial arm (synchronous or sequential boost) affected TB random errors. Hasan et al^23^ reported correlation between mean 3D TB setup errors for bony anatomy verification (3D *d*_bone_) and breast volume. Our study showed that TBs in Regions 1 (medial) and 4 (lateral) had smaller and larger TB systematic errors in the AP and LR directions, respectively. It is likely that there was less movement of medial breast tissue compared with bony anatomy and significant movement of lateral breast tissue, which may help explain these results. Hasan et al^23^ reported correlation of 3D *d*_bone_ with TB distance from the chest wall determined using planning CT (*n* = 27). Similarly, Topolnjak et al^24^ showed that the distance of the TB from the chest wall was correlated with the difference between TB setup errors for the chest wall and breast surface (*r* = 0.5, *p* = 0.034).

### Time to perform clip and bony anatomy match

The time for matching using clips (*t*_clips_) or bony anatomy (*t*_bone_) was significantly different at individual centres. For Centre A (kVCBCT), t_bone_ was less than *t*_clip_ because bone matching was automated using chamfer matching (XVI synergy, Elekta Ltd, Crawley, UK). For centres C, D and E, *t*_bone_ was greater than t_clip_, indicating that 2DkV imaging bony anatomy matching was less time efficient than using clips. The differences in time to match bony anatomy between centres using 2DkV imaging are unknown but may be a result of different observers.

### Clinical relevance

The IMPORT High trial protocol recommends clip verification and a 5-mm PTV isotropic margin for boost RT. We calculated that a 9–10 mm and 7–8 mm margin is required for laser setup and bony anatomy verification, respectively (Table 4). Larger margins are likely to increase PTV volume and the dose to normal breast tissue and the heart.^25^ Where possible, clip verification should be used; if this is not available, bony anatomy verification (CBCT or 2DkV) offers modest reduction in PTV volume compared with laser-only setup. For bony anatomy verification, we assumed an online protocol with no action level; if an action level or offline protocol is used, these margins may be greater. In addition, clips may reduce setup error for the whole breast RT (*Σ*_WB_); using bony anatomy as a surrogate for the whole breast, we found that *Σ*_WB_ was significantly smaller in all directions after clip setup compared with after laser setup (data not given). This implied that in a synchronous boost setting, clip setup would allow a whole-breast PTV margin reduction. Further work is required to quantify this reduction.

Association of patient- and treatment-related factors with TB setup errors suggest that individualization of treatment margins could be considered. Non-isotropic margins are not currently employed in breast RT. This work suggests that patient-specific margins and non-isotropic margins should be considered. It also suggests that some patients benefit more from clip-based verification compared with bony anatomy verification than others. If appropriate margins are applied, patients with large breasts or laterally located TBs will benefit from a greater reduction in the breast tissue irradiated if clips are used. Conversely, patients with smaller breasts or medially located tumours may benefit less from clip-based verification.

### Study limitations

This study assumed no significant difference among patient populations from the five different centres. Comparison of patient- and treatment-related factors between centres found small differences between centres in the number of clips and seroma visibility only. Centres B and E had significantly greater seroma visibility [patients with easily visible seroma: A, 22%; B, 38%; C, 17%; D, 13%; and E; 53% (*p* = 0.024)] and median number of clips [A, C and D, 6; B, 7; and E, 5 (*p* = 0.012)]. A large source of systematic error in breast boost RT, delineation error, has not been included in this analysis. Observer variation has been calculated in terms of the variation in TB volume (for example^17^); however, it is unclear how this will affect TB margins and there remains an opportunity for this to be explored. This work identifies the requirement for larger TB PTV margins if laser setup or bony anatomy verification is used, which results in a modest increase in the volume of normal breast tissue receiving the boost dose.^24^ The clinical effect of an increase in volume of normal tissue irradiated is not yet fully understood.^26^

## CONCLUSION

Patients with larger breasts, easily visible seroma and open surgical closing technique have greater setup errors when laser-only setup is used. Patients with larger breasts and laterally located tumours have greater setup errors when bony anatomy verification is used. If margins derived from patient setup errors are applied, these groups of patients will benefit from a greater reduction in breast tissue irradiated if clips are used. Clip verification enables smaller margins than bony anatomy verification and should be used where possible. If clips are not available, bony anatomy verification may give modest improvements in TB setup errors compared with laser setup, and individualization of TB margins may be considered based on breast volume, the position of the TB and seroma visibility.
